# Polychromatic Assessment of a Refractive Segmented EDOF Intraocular Lens

**DOI:** 10.3390/jcm11061480

**Published:** 2022-03-08

**Authors:** Scott García, Luís Salvá, Salvador García-Delpech, Anabel Martínez-Espert, Vicente Ferrando, Diego Montagud-Martínez

**Affiliations:** 1 Oftalmedic Salvá, 07013 Palma de Mallorca, Spain; brscottgarcia@gmail.com (S.G.); lsalva@oftalmedic.com (L.S.); 2 Fundación Aiken, 46004 Valencia, Spain; anabelmartinez@aikenval.com; 3 Departamento de Óptica, Optometría y CC de la Visión, Universitat de València, 46100 Valencia, Spain; diemonma@upvnet.upv.es; 4 Centro de Tecnologías Físicas, Universitat Politècnica de València, 46022 Valencia, Spain; viferma1@upv.es

**Keywords:** presbyopia correcting IOLs, EDOF IOLs, chromatic aberration, refractive segmented IOLs, optical bench

## Abstract

This study aimed to evaluate in vitro performance refractive segmented EDOF intraocular lenses under polychromatic light using an optical bench that complies with the ISO 11979-2 Norm. The through focus modulation transfer function (TF-MTF) of the Femtis Comfort LS-313 MF15 (Oculentis GmbH, Berlin, Germany) IOL was evaluated for IOLs with three different base powers. The effect of the asymmetry of the segmented designs was evaluated with 3 different wavelengths and with polychromatic light at a 3.0 mm and 5.0 mm pupil diameter. It was demonstrated that the TF-MTF curves exhibit a bifocal profile that, in practice, results in an EDOF design. As a consequence of the LCA, the TF-MTF values in white light were lower than in monochromatic light. Images of the USAF test chart were obtained to confirm the prediction of the TF-MTFs. We found that Femtis Comfort is a bifocal low-addition IOL and this fact can result in an EDOF effect which was obtained previously in clinical trials. Moreover, we showed that the base power influences the IOL optical quality, which results as more effective for high powers (hyperopic eyes) than for low powers (myopic eyes). The LCA of the segmented refractive design was very low and presumably not clinically relevant.

## 1. Introduction

The surgical options for the correction of presbyopia have increased significantly in recent years. In fact, new designs of intraocular lenses (IOLs) come to the market responding to the growing demand by patients for spectacle independence for vision at intermediate distances, where classic bifocals do not provide good vision. One approach is the so called extended depth of focus (EDOF) IOLs. The American Academy of Ophthalmology working group published a consensus statement for these IOLs: an EDOF is a lens that provides an extension of focus that is at least 0.50 D wider than for a monofocal IOL at a visual acuity of 0.2 logMAR [1]. There are several types of EDOF IOLs with different optical designs, most of them based on diffractive optics. The major shortcoming reported by users of diffractive IOL models is the incidence of disturbing visual phenomena like glare and halos, especially under mesopic illumination [2,3]. The alternative to diffractive IOLs are refractive designs which include rotationally symmetric designs and segmented designs [2]. The Femtis Comfort (Oculentis GmbH, Berlin, Germany) belongs to this last group. It was launched to the market as an EDOF IOL, unless it is in fact a bifocal IOL with low (1.50 D) addition value. According to several studies, this IOL has shown to be effective to improve patient’s visual performance for intermediate distances with minimal incidence of dysphotopsias [4,5,6,7,8]. One hypothesis for this good performance could be that, unlike what happens with diffractive designs in which chromatic aberration is a critical parameter [9,10,11,12,13] in refractive designs it is not so significant, since in this case only the Abbe Number and its refractive index influence its behavior under polychromatic light [14,15]. However, there is insufficient in vitro work with refractive multifocal lenses to validate this hypothesis. On the other hand, most of the recent comparative studies of the optical properties of IOLs are made with lenses of different models and with similar base power [11,12,16], but there is a lack of objective and experimental information about the influence of the lens power in refractive EDOF IOLs’ behavior under polychromatic illumination. In this respect the polychromatic performance of an IOL deserves attention since the increment in the power of the lens is accompanied by an increase in its thickness and/or radii of curvature affecting the optical path of light into the eye. In particular, to the best of our knowledge, the chromatic performance for different base powers of a segmented refractive IOL has never been objectively studied.

In this work we have performed the first comparative experimental study of the chromatic behavior of the Femtis Comfort IOL as a function of power, by using the through focus modulation transfer function (TF-MTF). Two different pupil diameters (corresponding to photopic and mesopic illumination conditions) were considered. Additionally, since this is a lens without revolution symmetry, we have investigated the differences between the TF-MTF along the sagittal and tangential meridians. These results have also not been previously reported in the literature. We studied the image quality provided by three different base power IOLs for different wavelengths separately (red, green and blue) and also with white light. Considering that we live in a polychromatic environment, the knowledge of the polychromatic response of every IOL in an optical bench is important to surgeons to know its main features and to predict its clinical outcomes.

## 2. Materials and Methods

### 2.1. Multifocal Intraocular Lenses

In this study, three different Femtis Comfort IOLs of base powers 15 D, 20 D, and 30 D were studied and compared. The Femtis Comfort IOL is a refractive lens with an optical zone of 5.7 mm that includes a rotationally asymmetric near vision segment of Add = 1.5 D. It is made of a copolymer consisting of hydrophilic acrylates with a hydrophobic surface. The optic is biconvex, with an aspheric posterior optic to neutralize high order aberrations. Additionally, it has a plate-haptic design with 4 flaps for capsulorhexis fixation.

**Figure 1:**
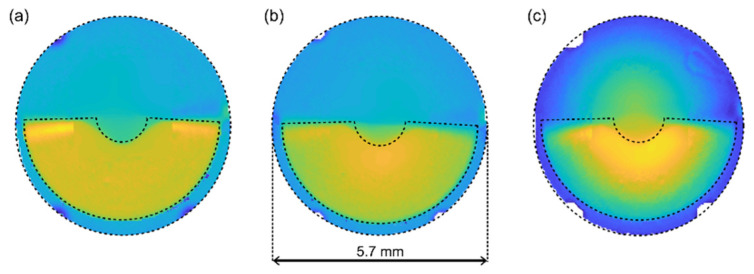
Power distribution in the Femtis Comfort lenses for 15 D (**a**), 20 D (**b**), and 30 D (**c**) base power. The blue and yellow areas indicate the labeled distance and near powers, respectively.

The relative phase profiles of the different lens base powers are shown in Figure 1. These images were obtained with a commercial NIMO TR1504 instrument (Lambda-X, Nivelles, Belgium). The far and near refractive zones can be clearly appreciated in blue and yellow, respectively. These measures are only qualitative and intended to show the zonal power distribution variation of the lens design.

### 2.2. Numerical Simulation

Numerical simulations of the expected experimental TF-MTF provided by a segmented refractive IOL emulating the Femtis Comfort profile were first performed in order to identify the expected differences between the tangential and sagittal profiles. To this end, we employed a commercial ray tracing optical design software (Zemax OpticStudio Design Software; version 18.7, LLC, Kirkland, WA, USA) in which all the elements of the optical bench were included. For this simulation we have considered a lens of 20 D base power and 1.5 D Add. Due to the lack of manufacturer’s data, we used typical values of other IOL models for the radii of curvature $r_{1}=21.4$ mm, $r_{2}=-8.73$ mm and thickness 0.7 mm. The Femtis Comfort profile was introduced as a “grid sag” surface in anterior face. The refractive index of the lens (n = 1.46) and the material Abbe Number (AN = 58) were available data from the manufacturer. The asphericity (Q) for the anterior segmented surface of the IOL was Q = 0, and for the posterior surface it was calculated to neutralize the spherical aberration of the lens as claimed for the manufactures, resulting Q = −3.51. The numerical simulations were performed under monochromatic green light (550 nm).

### 2.3. Optical Bench

**Figure 2:**
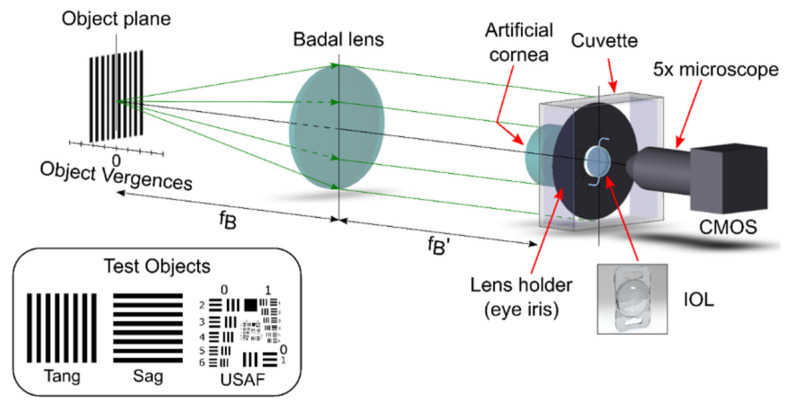
Optical bench. The test object can be illuminated by a monochromatic or polychromatic collimated beam (see the main text for details).

As detailed in an earlier publication [17], our experimental device is based on a custom-made image-forming setup. It meets the requirements of the International Organization ISO 11979-2, 2014 [18], and is able to obtain the polychromatic TF-MTF of a multifocal IOL in an automated procedure, controlled by a software programmed in LabView 2021 Professional^®^ (National Instruments, Austin, TX, USA). A representation of the optical setup is shown in Figure 2. The model eye consisted of an artificial cornea and a wet cell, filled with a saline solution, where the IOLs under test were placed in two different holders having different pupil sizes, with the Add segment at the bottom, as shown in Figure 1 and Figure 2. In this study, an achromatic lens with nearly zero spherical aberration (Melles Griot LA034 27.8 D, Thorlabs Inc., Newton, NJ, USA) has been used as a model cornea (ISO 1 cornea [18]) to avoid the mutual interaction between LCA and spherical aberration [19]. To obtain the image of formed by the artificial eye, a CMOS camera (EO-5012C; Edmund Optics, Barrington, NJ, USA) attached to an X5 microscope was used. The camera and the microscope were mounted on an XYZ translation stage to center the optical axis with the center of the lens and the artificial pupil. The illumination system (not shown in the figure) provided a collimated beam from a white LED (Thorlabs MCWHL5, Thorlabs Inc., Newton, NJ, USA), which, in this experiment, was sequentially filtered with 3 different chromatic filters of 10 nm bandwidth, each one centered at 450 nm, 550 nm and 650 nm (Thorlabs FB450-10; FB550-10; FB650-10, Thorlabs Inc., Newton, NJ, USA). The test object was mounted on a stepping motorized translation stage to generate the different vergences. It is important to note here that these vergences were measured from the object focal plane of an achromatic lens of 160 mm focal length (see the Badal lens, see Figure 2). Therefore, in our experimental setup, the angular size of the target images was independent of target position; therefore, IOLs with different powers do not produce significant variations in the final image magnification [17]. In this way, results obtained for 15 D, 20 D and 30 D base power IOLs can be directly compared.

To isolate polychromatic optical performance of the IOLs from the residual chromatic aberration generated by the optical elements in the optical bench, the optical setup was calibrated by measuring the LCA of the system without the IOL, and this result was subtracted from the experimental TF-MTF curves.

As detailed in Ref. [17], TF-MTFs at 50 lp/mm were obtained with a periodic binary grating acting as test object. Each MTF was obtained from the calculation contrast of the image registered by the CMOS camera. In this way, the system is capable of measuring the sagittal and tangential MTFs independently, simply by rotating the orientation of the test grating 90 degrees (see the inset in Figure 2). This feature is very important in this work to differentiate between the two perpendicular MTFs produced by the rotational asymmetry of the IOLs. Therefore, each measurement of the TF-MTF was obtained in both orientations and then the mean value of the sagittal and tangential counterparts was computed.

Finally, in order to evaluate the imaging performance of the IOLs in the model eye under polychromatic illumination, the test object has been replaced by the 1951 USAF resolution chart and the corresponding images were obtained at different vergences.

## 3. Results

### TF-MTF Curves. Numerical vs. Experimental Results

To realize the differences between tangential and sagittal TF-MTFs, the first step was to obtain numerically the characteristic tangential and sagittal TF-MTFs of the segmented refractive design for a single wavelength (550 nm), and then compare them with the experimental results in the optical bench.

**Figure 3:**
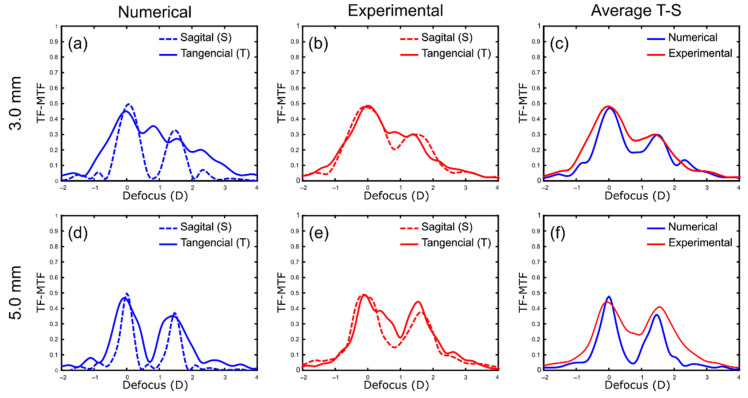
Numerically calculated (**a**,**d**) and experimentally obtained (**b**,**e**) monochromatic tangential and sagittal MTFs. The average MTFs, numerical (blue lines) and experimental (red lines), for 3.0 mm and 5.0 mm pupil diameters are shown in the graphs on the right (**c**,**f**).

Using data available from the Femtis Comfort, we performed the numerical simulation with Zemax in green light for a 20 D IOL base power. This merit function was computed for 3.0 mm and 5.0 mm pupil diameters. Figure 3a shows the numerical results, in which zeroth defocus corresponds to the far focus. Note the different profiles of the sagittal and tangential TF-MTFs. Interestingly, for both pupils the sagittal result is clearly bifocal, while the tangential one provides an extended depth of focus, especially for the 3.0 mm pupil (see Figure 3a). For the larger pupil, the contributions of the two segments of the IOL to the TF-MTFs are more balanced (Figure 3d), reinforcing the bifocal profile.

The corresponding experimental results are shown in the central column of Figure 3 (Figure 3b,e). Although the differences between the tangential and sagittal MTFs are attenuated with respect to the numerical prediction (Figure 3a,d), a good correspondence between numerical and experimental results have been obtained. In particular, note that for the 3.0 mm pupil, the peak of the tangential TF-MTF curves around 0.75 D (continuous lines) is present in both numerical and experimental results (Figure 3a,b). The above mentioned differences can be attributed to the unavailable data of the Femtis Comfort, needed for the exact numerical simulation (Q, radii of curvature, and lens thickness). Even if there is clear difference between sagittal and tangential TF-MTFs, especially for small pupil diameters, as natural scenes have in general a global view of spatial frequencies in different directions, what the eye sees with this lens implanted can be predicted by averaging the tangential and sagittal components of the MTF. Therefore, in the curves of the right column of Figure 3, the average of the tangential and sagittal TF-MTF have been calculated, ultimately confirming the good concordance between numerical and experimental results (see Figure 3c,f).

**Figure 4:**
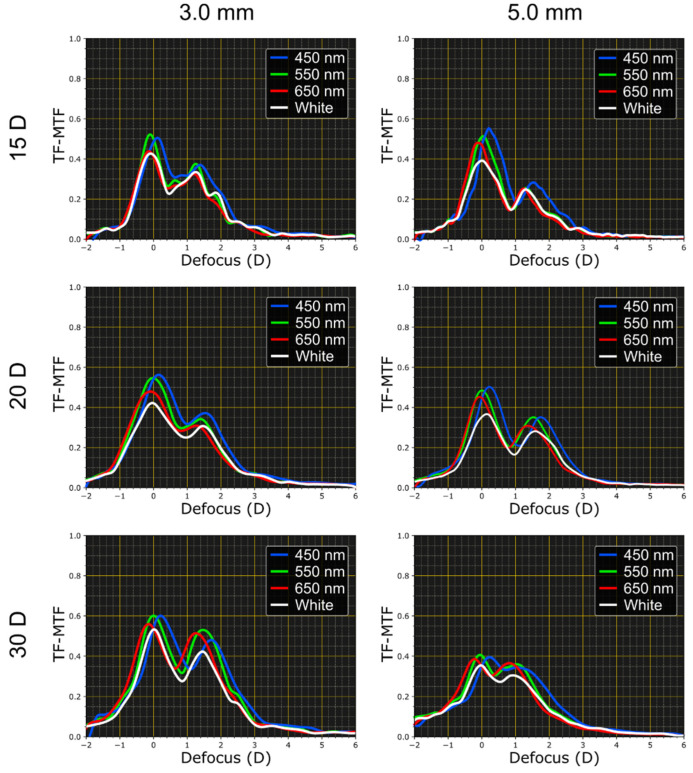
Polychromatic TF-MTFs of Femtis Comfort lenses of different base power.

Figure 4 shows experimental TF-MTF curves obtained for the Femtis Comfort IOLs of three different base powers with 3.0 mm and 5.0 mm pupil diameters, these results were obtained with 3 different wavelengths separated by 100 nm, and also with white light. In order to obtain comparable results, for each lens measures, the green far focus was taken as a reference and centered at the zeroth defocus. Measures for each wavelength and white light were obtained sequentially without moving any other element of the system, except for the chromatic filter located before the object plane. As expected, the order in which the peaks appear (red, green, blue) is associated with the refractive character of these IOLs in both the far and near foci. In fact, in the far and near foci, the red-maximum appears for lower power values and blue-maximum for higher values of defocus.

The low-addition bifocal nature of the Femtis Comfort design is verified for all wavelengths. This behavior is also qualitatively preserved for both pupil diameters in each lens power.

The TF-MTFs in Figure 4 show that the Femtis Comfort IOL is very low pupil-dependent but, as predicted in the numerical results in Figure 3c,f, has a better near focus for a small pupil. At this point, it is important to point out that the ISO Standards provide acceptable tolerance limitations for the manufacturers that vary with dioptric power ranges. The tolerance limits increase from ±0.3 D for IOL powers labeled less than 15.0 D, to ±1.0 D for IOL powers labeled greater than 30.0 D [18,20].

The LCA was obtained from the corresponding TF-MTF curves as the difference, in diopters, between the blue (450 nm) and the red (650 nm) focal planes. The results are shown in Table 1. The experimental error in each measurement of LCA is ±0.11 D.

**Figure 5:**
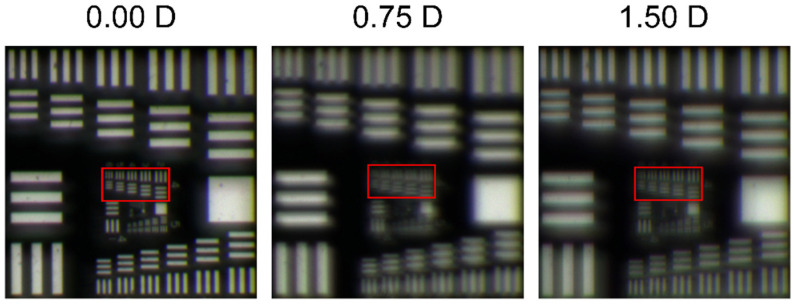
Experimental polychromatic USAF test images, at different vergences, obtained for a 30 D power lens with 3.0 mm pupil diameter.

Finally, Figure 5 shows the images, under white light illumination, of the USAF test obtained with the 30 D base power lens for a 3.0 mm pupil at different object vergences (0.0 D; 0.75 D and 1.5 D).

A very clear correspondence of these images with the TF-MTFs shown in Figure 4 can be observed. Several features should be remarked: First, the best image was obtained at the distance plane. Second, the quality of the image at the de second focus (1.5 D) has a little bit poorer resolution than the image at the distance plane. Thus, both images correspond qualitatively to the height of the two peaks in Figure 3 and Figure 4. Third, the image at the intermediate plane of 0.75 D coincides with the valley of the TF-MTFs in Figure 3 and Figure 4, confirming the bifocal nature of this lens. Finally, note also that the images of the central group of bars (red box) the vertical lines are more well focused than the horizontal ones at 1.50 D, but the opposite occurs at 0.75 D. Similar results were obtained for the other lenses of powers 15 D and 20 D.

## 4. Discussion

The purpose of this work was to provide objective evidence of the influence of the asymmetric design and different base powers on the polychromatic behavior of segmented refractive EDOFs. We have shown that the experimental TF-MTFs obtained independently in two perpendicular directions (sagittal and tangential) can be used to predict the image quality of these types of IOLs by averaging these two components. In this sense, we obtained evidence not previously mentioned neither in the literature, nor by the manufacturer itself, that the combination of two powers in different sectors of the lens causes an overlapping of the tangential and sagittal TF-MTFs that favors an extension of focus, especially for small pupil diameters. However, the astigmatic-like images that can be observed in Figure 5 in the high spatial frequency group of bars (red boxes) are a consequence of the different profiles of the sagittal ant tangential TF-MTFs presented in Figure 3.

Our results confirmed that the EDOF Femtis Comfort is actually a bifocal IOL with an Add value of 1.5 D. In fact, the TF-MTFs we obtained for different powers and pupil diameters all showed a bifocal profile. This conclusion can also be obtained from the USAF test images (see Figure 5) since the images at 1.50 D and 0.0 D are globally better than the image at 0.75 D. Therefore, the EDOF effect in this lens is achieved by the partial overlapping of two nearby foci.

A recent clinical study that reported outcomes of the Femtis Comfort [21] confirms that this IOL offers a very good functional range of vision and minimal unwanted visual phenomena. In fact, another objective of having a smaller addition was to reduce the “angel wing glare effect”, reported for other IOL models with the same design, but with a larger Add value [22].

We found the LCA of the Femtis Comfort in both foci is positive, as expected for a pure refractive design, although the amount of the LCA measured in all cases was lower than 0.5 D and no significant differences were obtained in the LCA for photopic and mesopic pupil diameters. Nevertheless, the TF-MTF values in white light were lower than those obtained with monochromatic light. The influence of the lens power on chromatic aberrations was numerically studied in eye models by Song et al. [23] working with monofocal IOLs. They found that the MTF values increased with the IOL power, under both monochromatic and polychromatic light, and they suggested that this effect could be caused by the interactions of monochromatic aberrations and chromatic aberrations in the eye model. This finding is consistent with our results since in our experiment, the LCA of the model eye was neutralized. Moreover, similar to the present work, they found that, as a consequence of the LCA, the MTF values were significantly lower when measured under polychromatic illumination than those measured with monochromatic light.

In another related work, Radhakrishnan et al. [24] measured the subjective visual performance of the same design IOL under different orientations and found that perceptual performance was different across orientations and distances in most subjects. They concluded that these preferences were associated with the optical quality of each eye defined by its low and high order aberrations. In this work, we demonstrated that the lens design could also play a role in this respect (see Figure 5). Therefore, although the results in this study cannot be directly extrapolated to clinical expectation because the image quality of the eye under polychromatic light is influenced by other factors (mostly eye-specific factors), findings from this study provide insight about the optical properties of sectorial multifocal designs that could be correlated with results obtained in clinical studies. Moreover, other important clinical issues, such as the effect of the lens tilt and decentration should be evaluated numerically and in vitro to complement this information.

## 5. Conclusions

In conclusion, to the best of the authors’ knowledge, this is the first study that reports experimental objective metrics for the Femtis Comfort EDOF MIOL with polychromatic light. We measured the sagittal and tangential TF-MTF for 3 different base power lenses under different wavelengths and white light, and two pupil diameters. We demonstrated that additionally to the low Add value, the segmented bifocal design gives the lens its EDOF character. In fact, we have shown that the image quality of this type of segmented EDOF can be explained by means of the average of the sagittal and tangential TF-MTFs.

Despite the base power influencing the optical quality of this refractive EDOF IOL, the LCA was lower than 0.5 D independently of the base power and pupil diameter. Therefore, in the Femtis Comfort, the LCA presumably is not clinically relevant.

## Figures and Tables

**Table 1:** LCA (D) of Femtis Comfort IOLs measured at the far and distance foci.

Base Power	15 D		20 D		30 D	
Pupil diameter	3.0 mm	5.0 mm	3.0 mm	5.0 mm	3.0 mm	5.0 mm
0.0 D	0.26	0.31	0.25	0.29	0.34	0.54
1.5 D	0.20	0.29	0.25	0.34	0.30	0.49

## Data Availability

The data that support the findings of this study are available from the corresponding author, S.G.-D., upon reasonable request.
